# Emergent wave phenomena in coupled elastic bars: from extreme attenuation to realization of elastodynamic switches

**DOI:** 10.1038/s41598-017-16364-8

**Published:** 2017-11-24

**Authors:** Qianli Chen, Ahmed Elbanna

**Affiliations:** Department of Civil and Environmental Engineering, University of Illinois at Urbana, Champaign, USA

## Abstract

*Metamaterials* with acoustic and elastic band gaps are of great interest to scientists and engineers. Here, we introduce a novel mechanism for emergence of multiple band gaps with extreme attenuation by coupling continuous one-dimensional elastic structures. We show that it is possible to develop extreme attenuation at several frequencies from coupling two homogenous bars of different elastodynamic properties even though each bar individually possesses no such gaps. Moreover, if each bar is a composite on its own, multiple resonant band gaps appear in the compound system which do not exist in either bar. We verify our results by conducting numerical simulations for the elastodynamic response and show that the resonant gaps are efficient in attenuating wave propagation. Furthermore, we show that by carefully tailoring the properties of the coupled bars we may construct elastodynamic signal choppers. These results open a new gate for designing *Metamaterial* with unique wave modulation properties.

## Introduction

Classical elastic composite materials exhibit mechanical properties constrained by their constituents. By careful design, the composite may have optimized combination of mechanical properties^1^ but the upper and lower boundary of effective properties cannot exceed the Hashin Shtrikman limits^2,3^. In the past two decades, the field of *Metamaterials* has attracted much attention. *Metamaterials* possess extreme material properties not common in nature and those properties are usually derived from geometry and topology rather than the properties of the constituents^4,5^. The concept of a *Metamaterial* originates in the study of manipulating electromagnetic waves^6,7^ and was later brought to acoustic^8,9^ and elastic^10–12^ waves due to the similarity of the mathematical structure^13^. Negative permittivity and permeability may be achieved for electromagnetic waves while negative density and elastic moduli may be achieved in the context of elastic waves. With the modification of Newton’s second law^14^, the periodic elastic composite may be homogenized and designed to exhibit frequency dependent effective properties^15,16^. Those effective properties lead to efficient wave manipulation and result in applications such as enhanced wave damping^17^, wave guides^18^, noise filters^19^ and cloaking^20,21^.

A special property of *Metamaterials* is the presence of band gaps^15,16,22–26^. A band gap refers to a frequency range where wave cannot propagate. Early findings about band gap mechanisms suggest they emerge due to the Bragg diffraction. Waves interact with super and sub-wavelength microstructure, through constructive and destructive interference, leading to the development of a band gap when the wavelength is comparable to the structure periodicity. Recent research suggests local resonance^27,28^ to be an alternative/complementary mechanism which enables generating much lower band gaps compared to the diffraction gaps emerging from the microstructure periodicity. Usually this is achieved by coupling the structure to discrete attachments, typically a spring-mass system, to generate a single band gap at the frequency corresponding to the natural frequency of the attachment. The attached spring mass system resonates at that frequency and vibrates at increased amplitude shielding the main system from the wave effect. In this paper, we introduce a novel way to generate multiple resonant gaps by periodically coupling two elastic systems in parallel. We focus primarily on one-dimensional (1D) elastic bars but extension to higher dimensions is straightforward.

## Results

### Dispersion relation

**Figure 1:**
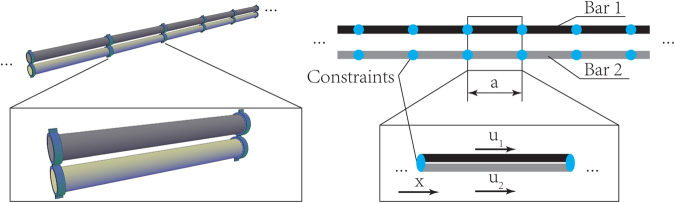
A schematic of two bars in parallel coupled periodically using rigid couplers (with period = *a*) and the representative unit cell. Couplers are portrayed for illustration purposes only. We propose different alternatives for coupling in the discussion section.

We consider our basic system as two bars connected in parallel as shown schematically in Fig. 1. Each bar is a periodic structure with periodicity *a*. The two bars are connected at discrete locations (marked by the blue dots) with the same periodicity *a*. Those periodic connections are assumed to be perfect bonds at which the displacement of the two bars is constrained to be the same.

We look for travelling wave solutions of the elastodynamic equations in this periodic system. Specifically, we seek to find for a given frequency/wave number, what the admissible corresponding wavenumber/frequency solutions are. The relation between the two pairs is called the dispersion relation. The analytical expression for the dispersion relation of the two bars coupled in parallel is given by: [See Methods for the full derivation]

1 $$\cos (qa)=\frac{{z}_{1}\,\cos ({\theta }_{1})\sin ({\theta }_{2})+{z}_{2}\,\sin ({\theta }_{1})\cos ({\theta }_{2})}{{z}_{1}\,\sin ({\theta }_{2})+{z}_{2}\,\sin ({\theta }_{1})}$$

where ${\theta }_{i}=\omega a/{c}_{i}$ is the normalized frequency, ${z}_{i}={\rho }_{i}{c}_{i}$ is the impedance and the bar index $i=1,2$.

**Figure 2:**
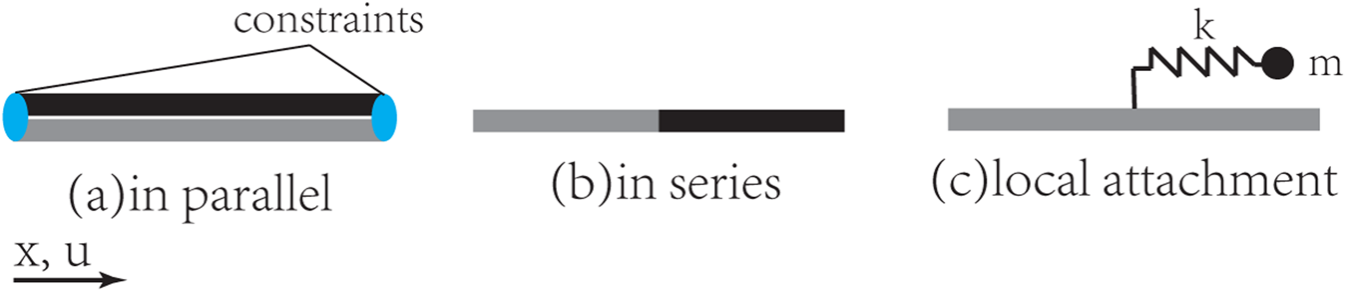
The representative unit cells for different composite systems [gray and black represent two different materials]. (**a**) Two material components coupled in parallel. (**b**) Two material components coupled in series. (**c**) A single material component is coupled with a spring mass system.

The dispersion relation of a periodic composite bar with two material components in series (Fig. 2b) is given by Rytov^29^ as below

2 $$\cos (qa)=\,\cos ({\theta }_{1})\cos ({\theta }_{2})-\frac{1}{2{z}_{1}{z}_{2}}({z}_{1}+{z}_{2})\sin ({\theta }_{1})\sin ({\theta }_{2})$$

where ${\theta }_{i}\,=\omega {L}_{i}/{c}_{i}$, ${L}_{i}$ is the length of for material $i=1,2$.

A quotient dispersion relation, of a different form from equation (1), is also found in elastic systems with locally resonant spring-mass attachment^30^ as

3 $$\cos (qa)=\,\cos (\theta )-\frac{\omega mk}{2z({\rm{k}}-{\omega }^{2}{\rm{m}})}\,\sin (\theta )$$

where a mass *m* is attached to a homogeneous bar with spring of stiffness *k* in periodicity of *a*. The bar has the density per length $\rho$, wave speed *c*, acoustic impedance $z=\rho c$, and $\theta \,=\omega a/{\rm{c}}$ as shown in Fig. 2c. The denominator on the right-hand side (RHS) of equation (3) becomes zero when $\omega ={\omega }_{0}=\sqrt{k/{\rm{m}}}$, i.e. the resonant frequency of the attached spring mass system. This results in a single locally resonant band gap.

When ${c}_{1}\ne {c}_{2}$ and ${\theta }_{1}\ne {\theta }_{2}$, the denominator of right hand side of equation (1) may become zero which results in singularity points in the dispersion relation. Those points, similar to the local resonant frequencies in equation (3), give infinite imaginary part of the corresponding wave number. But unlike the single resonant frequency in equation (3), the denominator in the right-hand side of equation (1) yields infinite countable resonant frequencies. When *c*_2_/*c*_1_ is a rational number other than 1, the denominator of the RHS of equation (1) is a periodic function. Since the solutions of $\omega$ when the denominator equals to zero are the resonant frequencies, the resonant frequencies are periodic in this case. When *c*_2_/*c*_1_ is an irrational number, the denominator becomes quasi-periodic and so do the resonant frequencies.

### Coupling of homogeneous bars

In the following examples, we choose a homogeneous bar 1 with reference material of ${\rho }_{1}=1$, ${(EA)}_{1}=1$ and wave speed ${c}_{1}=\sqrt{{(EA)}_{1}/{\rho }_{1}}=1$. We couple this bar periodically ($a=1$) with another homogeneous one with a generally different wave speed ${c}_{2}\ne {c}_{1}$. The dispersion relation of the coupled system is given by equation (1).

**Figure 3:**
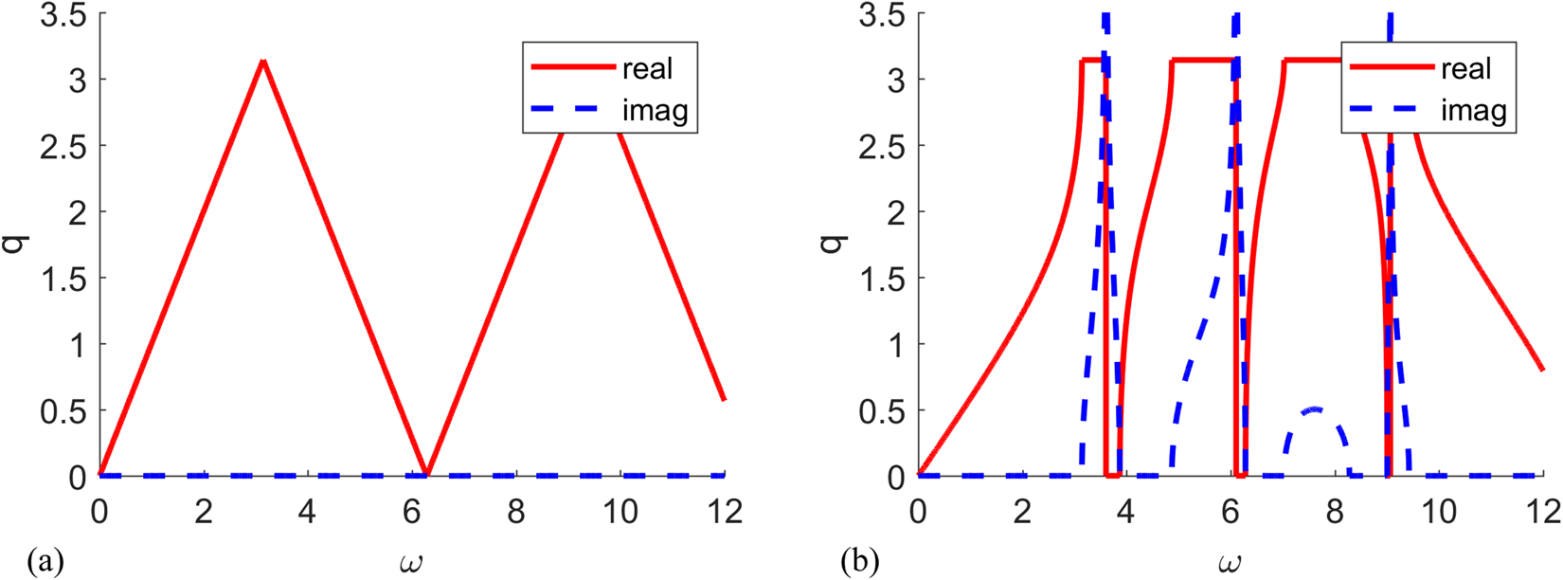
Dispersion relation of coupled parallel homogeneous bars (**a**) linear dispersion relation with $a=1$, ${\rho }_{1}={\rho }_{2}/5=1$, ${(EA)}_{1}={(EA)}_{2}/5=1$, ${c}_{1}={c}_{2}$ (**b**) dispersion relation with resonant frequencies $a=1$, ${\rho }_{1}={\rho }_{2}=1$, ${(EA)}_{1}={(EA)}_{2}/5=1$, ${c}_{1}\ne {c}_{2}$.

The dispersion relation shows distinct behavior depending on the relative magnitudes of the wave speeds *c*_2_ and *c*_1_. When *c*_2_ = *c*_1_, the dispersion relation is of the same form of a single homogeneous bar (Fig. 3a). This is even though the two bars have materials with different densities and elastic properties (${\rho }_{1}={\rho }_{2}/5=1$, ${(EA)}_{1}={(EA)}_{2}/5=1$). When ${c}_{2}\ne {c}_{1}$, the solution for the wave numbers become complex for certain frequencies and band gaps appear. A case with ${\rho }_{1}={\rho }_{2}=1$, ${(EA)}_{1}={(EA)}_{2}/5=1$ is shown in Fig. 3b. Within the frequency range from 0 to 12, there are four band gaps at $\omega$ within $[3.14,3.87]$, $[4.87,6.28]$, $[7.02,8.28]$ and $[9.01,9.43]$. Other than the third band gap, all the band gaps include resonant frequencies at $\omega =3.60,6.10,9.06$ respectively.

To study the elastodynamic response of the periodic structure, we conduct a Finite Element (FE) simulation of the parallel bars with 50 unit cells of periodicity $a=1$. At the left end, we prescribe a displacement ${u}_{left}=\,\sin (\omega t)$ with $\omega$ the excitation frequency and t the time. The right end is fixed and the simulation is ended when the wave front reaches the right end to exclude any far field boundary effects.

**Figure 4:**
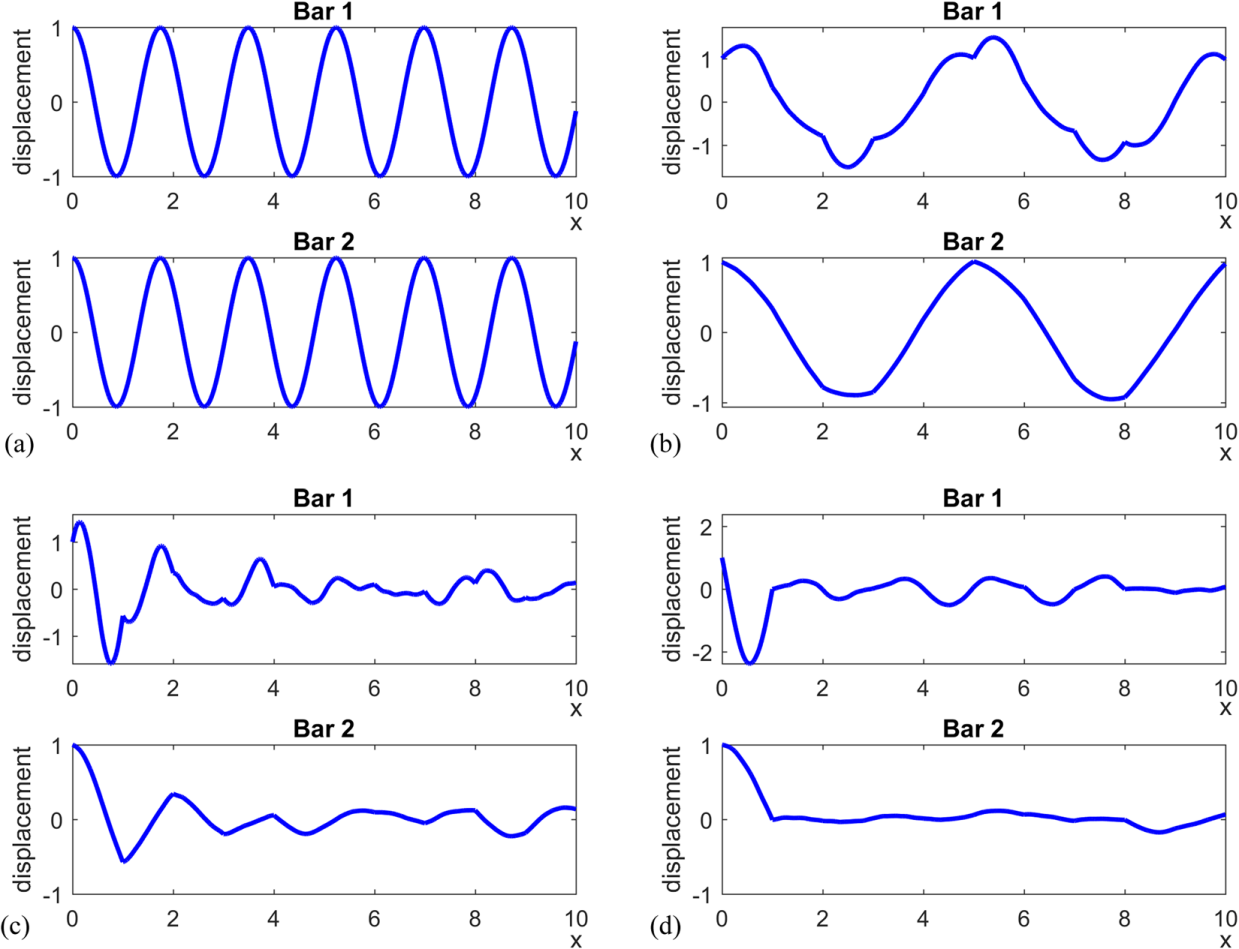
Snapshots of the elastodynamic response of the coupled parallel homogeneous bars with (**a**) same wave speed, and (**b**)–(**d**) different wave speeds. Simulation parameters for (**a**) are $\omega =3.60$, $a=1$, ${\rho }_{1}={\rho }_{2}/5=1$, ${(EA)}_{1}={(EA)}_{2}/5=1$. Simulation parameters for the other cases are $a=1$, ${\rho }_{1}={\rho }_{2}=1$, ${(EA)}_{1}={(EA)}_{2}/5=1$ with excitation characteristic given by (**b**) $\omega =2$, $q={\rm{1.23}}$ (outside band gap) (**c**) $\omega =5$, $q=\pi +0.52i$ (inside band gap) and (**d**) $\omega =3.60$, $q=\pi +{\rm{5.17}}i$ (the resonant frequency within the first band gap).

Snapshots of the response of the first 10 unit cells are plotted in Fig. 4. In Figure 4a, when ${\rho }_{1}={\rho }_{2}/5=1$ and ${(EA)}_{1}={(EA)}_{2}/5=1$ so that ${c}_{1}={c}_{2}$, as indicated by the linear dispersion relation in Fig. 4a, the wave propagates without dispersion. With the same wave speed in both bars, the two bars behave in exactly the same way and the periodic coupling constraint is not active. When ${\rho }_{1}={\rho }_{2}=1$, ${(EA)}_{1}={(EA)}_{2}/5=1$ I and ${c}_{1}\ne {c}_{2}$, the response of the two bars becomes distinct due to dispersion effects as shown in Fig. 4b–d. The wave propagation is dependent on whether the excitation frequency falls inside or outside the band gaps. When $\omega =2$, a frequency outside band gap, the corresponding wavenumber is $q={\rm{1.23}}$ and the wave propagates without changing of magnitude as shown in Fig. 4b. When $\omega =5$, a frequency within the band gap, the corresponding wavenumber is complex, $q=\pi +0.52i$, indicating spatial attenuation as shown in Fig. 4c. When $\omega =3.6$, a resonant frequency within the band gap, the corresponding wave number is $q=\pi +{\rm{5.17}}i$. In this case, the wave decays faster with distance from the excitation source (left end).

### Coupling of composite bars

**Figure 5:**
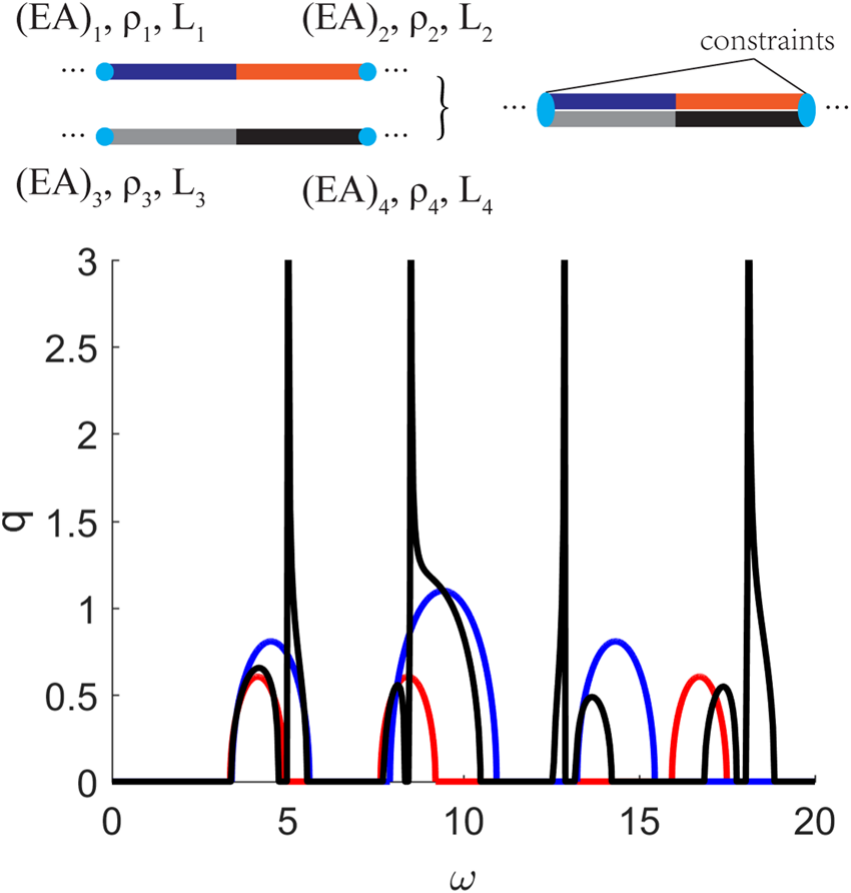
Imaginary part of the solution of dispersion relation (bottom) and unit cells (top) of two-phase composite bars and their coupling [red: $a=1$, ${\rho }_{1}={\rho }_{2}=1$, ${(EA)}_{1}={(EA)}_{2}/4=1$, ${L}_{1}={L}_{2}=a/2=0.5$; blue: $a=1$, ${\rho }_{3}={\rho }_{4}=1$, ${(EA)}_{3}={(EA)}_{4}/9=1$, ${L}_{3}={L}_{4}=a/2=0.5$; black: with coupling of these two in periodicity of $a=1$].

We consider one composite bar of periodic unit cell made of two materials with $a=1$, ${\rho }_{1}={\rho }_{2}=1$, ${(EA)}_{1}={(EA)}_{2}/4=1$, ${L}_{1}={L}_{2}=a/2=0.5$ and another one with $a=1$, ${\rho }_{3}={\rho }_{4}=1$, ${(EA)}_{3}={(EA)}_{4}/9=1$, ${L}_{3}={L}_{4}=a/2=0.5$ as shown in Fig. 5. Following the same procedure outlined in the Methods section, we derive the dispersion relation corresponding to the coupled composite bars in equation (4). The imaginary part of the solution for this dispersion relation is shown in Fig. 5 along with the corresponding solution for each bar individually. The combination of the two bars results in 8 band gaps ([3.3, 4.7], [5.0, 5.6], [7.7, 8.3], [8.5, 10.5], [12.6, 12.9], [13.2, 14.2], [16.9, 17.8], [18.1, 18.9]) within the same frequency domain. Since the dispersion relation shown in equation (4) is a quotient, there are 4 resonant frequencies $\omega =5.02,8.50,12.88,18.12$ between 0 and 20. We also note that Fig. 5 suggests the band gaps in the coupled system need not be coincident with the band gaps in individual bars, but overlaps exist between the two, especially for the lower frequency band gaps. This suggests that the band structure for the coupled bars may cover frequency ranges that are not covered by either bar individually. It also shows the strong effect of coupling which leads to extreme attenuation at several frequencies while the band gaps in either bar have a bounded imaginary wave number solution and thus bounded attenuation.

4 $$\begin{array}{rcl}\cos (qa) & = & \{{z}_{1}{z}_{2}{z}_{3}\,\cos ({\theta }_{1})\cos ({\theta }_{2})\cos ({\theta }_{3})\sin ({\theta }_{4})+{z}_{1}{z}_{2}{z}_{4}\,\cos ({\theta }_{1})\cos ({\theta }_{2})\cos ({\theta }_{4})\sin ({\theta }_{3})\\  &  & +\,{z}_{1}{z}_{3}{z}_{4}\,\cos ({\theta }_{1})\cos ({\theta }_{3})\cos ({\theta }_{4})\sin ({\theta }_{2})+{z}_{2}{z}_{3}{z}_{4}\,\cos ({\theta }_{2})\cos ({\theta }_{3})\cos ({\theta }_{4})\sin ({\theta }_{1})\\  &  & -\,1/2[({z}_{1}{z}_{3}^{2}+{z}_{1}{z}_{4}^{2})\cos ({\theta }_{1})\sin ({\theta }_{2})\sin ({\theta }_{3})\sin ({\theta }_{4})+({z}_{1}^{2}{z}_{3}+{z}_{2}^{2}{z}_{3})\sin ({\theta }_{1})\sin ({\theta }_{2})\cos ({\theta }_{3})\sin ({\theta }_{4})\\  &  & +\,({z}_{2}{z}_{3}^{2}\,+\,{z}_{2}{z}_{4}^{2})\sin ({\theta }_{1})\cos ({\theta }_{2})\sin ({\theta }_{3})\sin ({\theta }_{4})+({z}_{1}^{2}{z}_{4}+{z}_{2}^{2}{z}_{4})\sin ({\theta }_{1})\sin ({\theta }_{2})\sin ({\theta }_{3})\cos ({\theta }_{4})]\}\\  &  & /[{z}_{1}{z}_{3}{z}_{4}\,\cos ({\theta }_{1})\sin ({\theta }_{2})+{z}_{2}{z}_{3}{z}_{4}\,\cos ({\theta }_{2})\sin ({\theta }_{1})+{z}_{1}{z}_{2}{z}_{3}\,\cos ({\theta }_{3})\sin ({\theta }_{4})+{z}_{1}{z}_{2}{z}_{4}\,\cos ({\theta }_{4})\sin ({\theta }_{3})]\end{array}$$

where $${\theta }_{i}\,=\omega {L}_{i}/{c}_{i},i=1,2,3,4$$.

### Effect of compliant coupling

**Figure 6:**
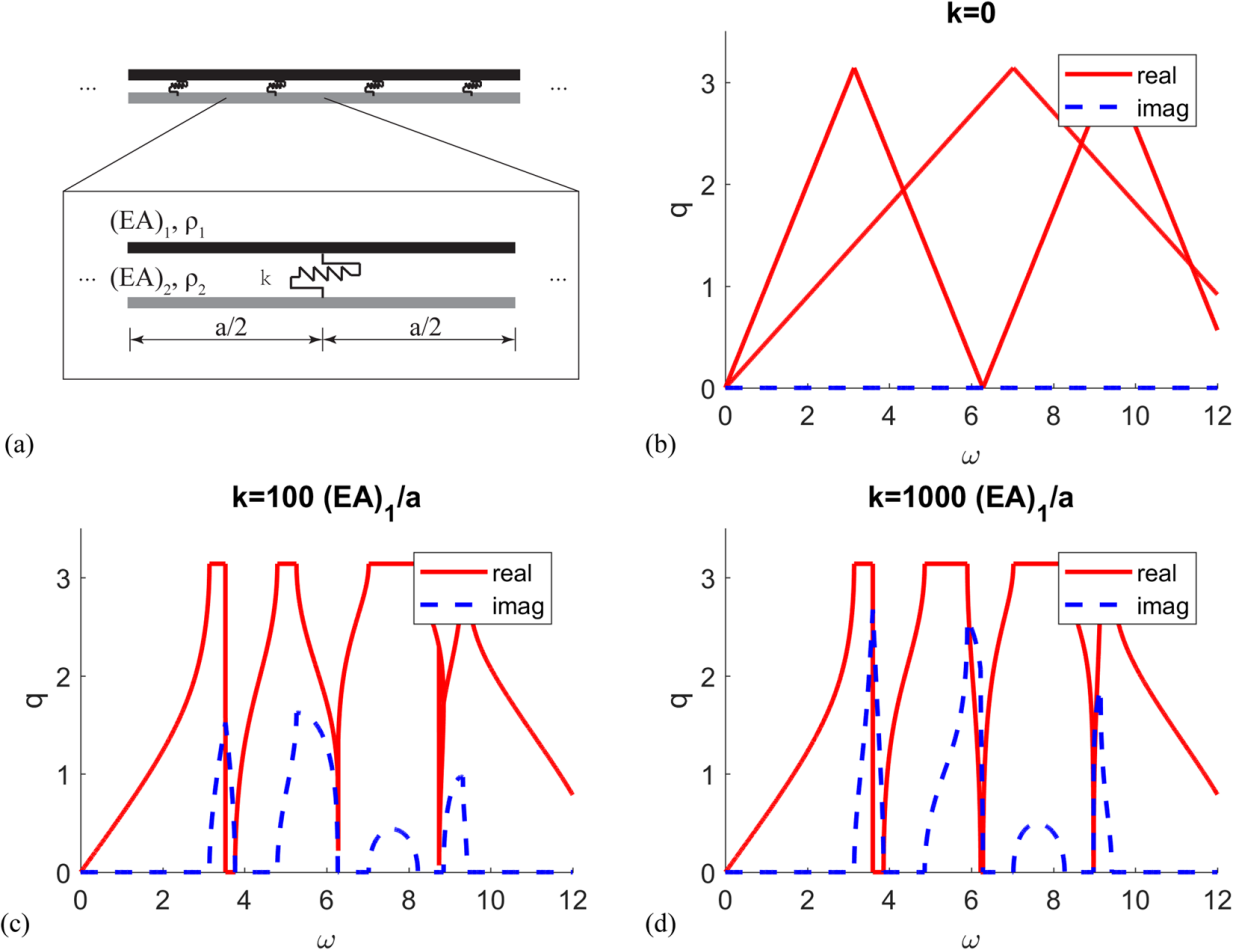
Dispersion relation and unit cells (**a**) of coupled parallel homogeneous bars with finite stiffness coupling (**b**) coupling stiffness $k=0$ (**c**) coupling stiffness $k=100{(EA)}_{1}/a$ (**d**) coupling stiffness $k=1000{(EA)}_{1}/a$ [$a=1$, ${\rho }_{1}={\rho }_{2}=1$, ${(EA)}_{1}={(EA)}_{2}/5=1$].

In practice, the two bars may not be perfectly coupled using rigid connections that enforce equal displacements of both bars at their common points. Here we explore how the relaxation of the rigidity constraint influences the band gap response. Assuming the coupling constraint to have a finite stiffness k (Fig. 6a), the dispersion relation of the two periodically coupled bars is given by [See Methods for details]:

5 $$\begin{array}{c}k({z}_{1}\,\sin ({\theta }_{2})+{z}_{2}\,\sin ({\theta }_{1}))\{\frac{{z}_{1}\,\cos ({\theta }_{1})\sin ({\theta }_{2})+{z}_{2}\,\sin ({\theta }_{1})\cos ({\theta }_{2})}{{z}_{1}\,\sin ({\theta }_{2})+{z}_{2}\,\sin ({\theta }_{1})}-\,\cos (qa)\}\\ \quad +\,2{z}_{1}{z}_{2}\omega [\cos (qa)-\,\cos ({\theta }_{1})][\,\cos (qa)-\,\cos ({\theta }_{2})]=0\end{array}$$

where $${\theta }_{i}=\omega a/{c}_{i},i=1,2$$.

If $k=0$, which corresponds to uncoupled bars, the first term vanishes, and the second term gives the linear dispersion relation of the two individual bars (Fig. 6b). If *k* is a finite number (Fig. 6c and Fig. 6d), the attenuation strength (measured by the imaginary part of the wavenumber) depends on the value of *k*. In the limit as $k\to \infty$, which corresponds to the perfect coupling case, the first term in equation (5) dominates and we recover the dispersion relation given by equation (1) with the imaginary parts of the wave number diverge at multiple resonant frequencies.

### Wave modulation

**Figure 7:**
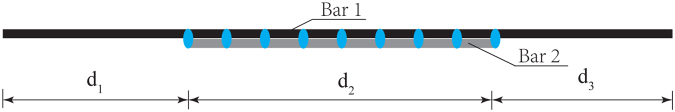
Simple device for signal modulation where a second bar (Bar 2) is coupled with the original through-going bar (Bar 1) over a finite length d_2_.

**Figure 8:**
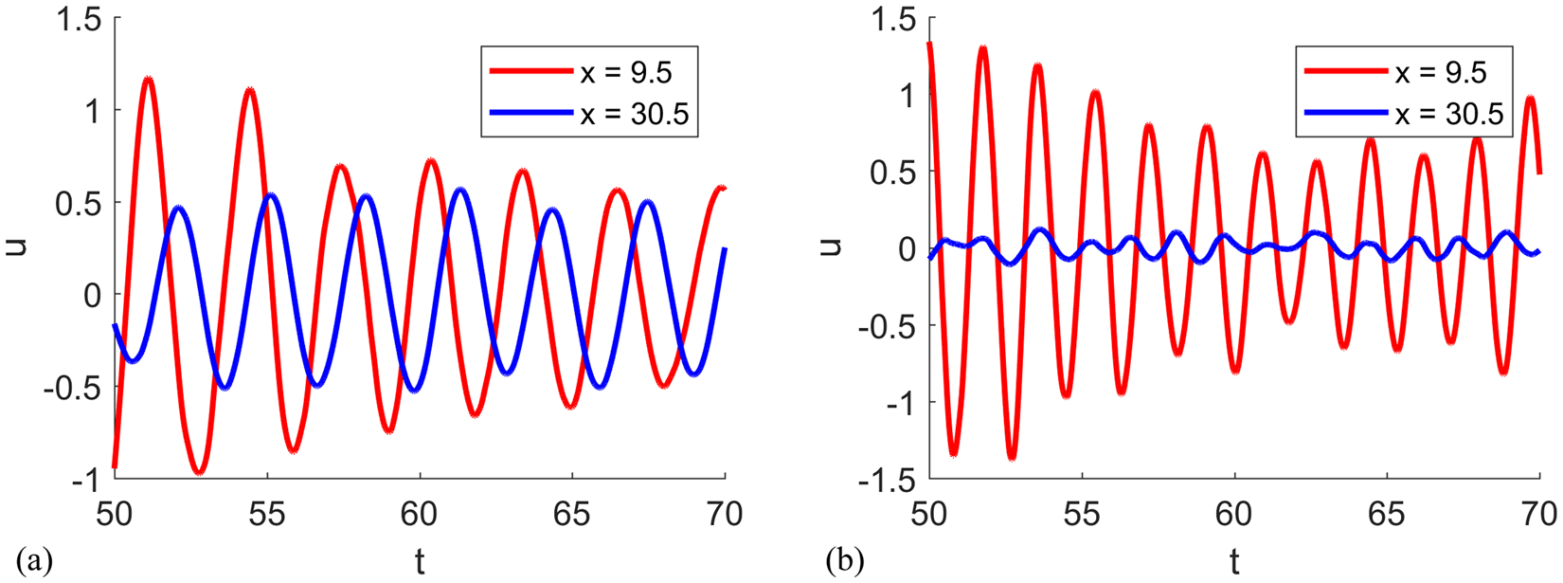
Response of the through-going bar to an excitation frequency (**a**) $\omega =2$ (**b**) $\omega =3.6$ at two different points (just before and after the coupling interval that extends between x = 9.5 and x = 30.5).

Motivated by the extreme attenuation exhibited by the coupled bars, we demonstrate a simple device for signal modulation as shown in Fig. 7. Bar 1 is homogeneous and thus possesses a linear dispersion relation with no band gaps if considered individually. With bar 1 alone, any signal propagates without attenuation. If we couple the homogeneous bar with another homogeneous bar of a different material over the interval $x\in [{{\rm{d}}}_{1},{{\rm{d}}}_{1}+{{\rm{d}}}_{2}]$, this part of the coupled bar represents a finite length realization for the periodic structure discussed in the previous section and is expected to exhibit band-gap like response for certain frequencies. As an example, we choose ${\rho }_{1}={\rho }_{2}=1$, ${(EA)}_{1}={(EA)}_{2}/5=1$, and $a=1$. In the device shown in Fig. 7, we set ${{\rm{d}}}_{1}=10$, ${{\rm{d}}}_{2}=20$, and ${{\rm{d}}}_{3}=50$. In this case, when displacement ${u}_{left}=\,\sin (\omega t)$ is applied to the left end, the wave propagates through d_1_ and gets modulated between $x\in [{{\rm{d}}}_{1},{{\rm{d}}}_{1}+{{\rm{d}}}_{2}]$ and outputs a new signal after $x={{\rm{d}}}_{1}+{{\rm{d}}}_{2}$. By comparing steady response of $\omega =2$ and $\omega =3.6$ at d_1_ and ${{\rm{d}}}_{1}+{{\rm{d}}}_{2}$ shown in Fig. 8, it is evident that for $\omega =3.6$, a frequency within the band gap, the response is largely reduced compared to the case of $\omega =2$, a frequency outside band gap, for which the response is much larger. This demonstrates that coupling two bars just over a finite length may have a strong effect on the elastodynamic response of the continuous through-going bar.

### Mechanical switch

Another unexpected phenomenon that becomes possible through coupling different elastic bars is the realization of mechanical switches or choppers. To see this, recall equation (1), where resonance occurs when the denominator is zero

6 $${z}_{1}\,\sin ({\theta }_{2})+{z}_{2}\,\sin ({\theta }_{1})=0$$

**Figure 9:**
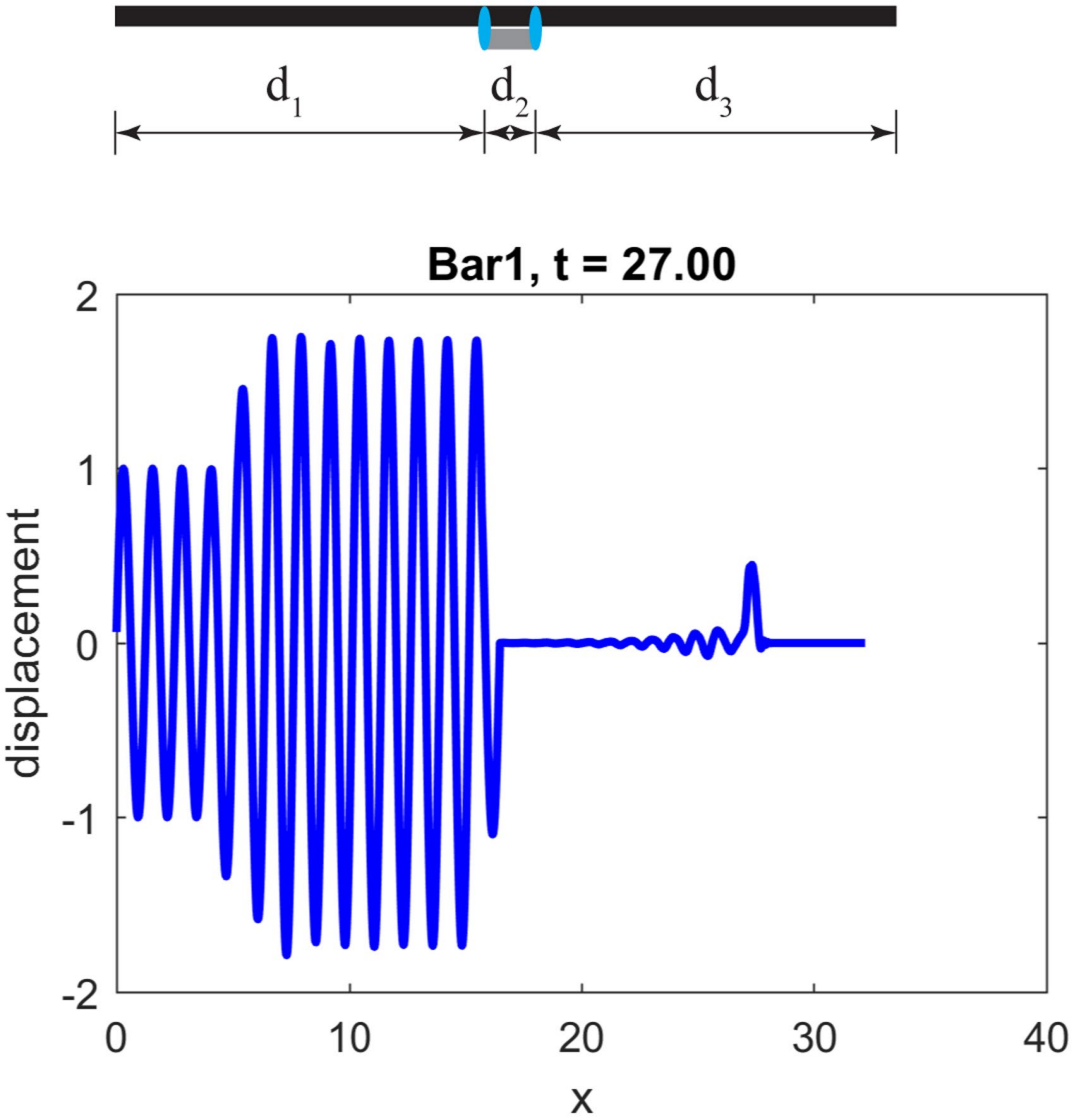
Response of partially coupled bars. (top) Schematic of the coupled system: a short bar of length *a*, and wave speed *c*_2_ is connected to a through-going bar of wave speed *c*_1_. (bottom) Snapshot of the dynamic response of the through-going bar at a time after which the input signal has passed through the coupling region showing that only the leading half sine wave of the signal persists.

To further simplify this case, we consider two materials with the same impedance so that equation (6) reduces to $\sin ({\theta }_{2})+\,\sin ({\theta }_{1})=0$. The family of solutions to this equation is ${\theta }_{1}={\theta }_{2}+\pi$ which gives $a/{c}_{1}=a/{c}_{2}+\pi /\omega$. This means that the time for a wave passing one spatial periodicity $a/{c}_{1}$ in the first bar is different by an amount $T/2=\pi /\omega$ relative to the time for a wave to pass $a/{c}_{2}$ in the other bar, where *T* is the period of the signal of interest. In this case, we show that even coupling the two bars over a single period *a* may annihilate the advancing wave through the continuous bar except for the first leading half sine wave. This occurs because the waves in the two bars will possess identical form (since the two bars have the same impedance) but with a 90° phase shift. To illustrate this, we choose ${(EA)}_{1}={(EA)}_{2}/5=1$, ${\rho }_{1}=5{\rho }_{2}=1$ so that ${z}_{1}={z}_{2}=1$, but ${c}_{1}={c}_{2}/5=1$ and $a=0.7854$ so that $a/{c}_{1}=a/{c}_{2}+\pi /\omega$, where we have chosen an arbitrary frequency $\omega =5$. With these material properties and a setup of the device shown in Fig. 9 with ${{\rm{d}}}_{1}={{\rm{d}}}_{3}=20a$ and ${{\rm{d}}}_{2}=a$ (i.e. coupling over an interval of only a single unit cell), the response after the wave has passed through the left junction point is shown. Except for some initial transient response (essentially the first half sine wave and some noise) passing before steady state, the rest of the signal is eliminated. This is a realization of a mechanical chopper that may be used to modulate mechanical signals, analogous to their electronic counterparts, provided the geometry and material properties of the attached short bar are properly selected according to the target frequency.

## Discussion

The resonant gap is usually produced by periodically attaching or incorporating a spring mass system to a macro structure. The resulting composite structure exhibits extreme attenuation at a single frequency given by the natural frequency of the mass-spring system signaling a local resonance effect. In this paper, we introduce a new way to create multiple resonant gaps by coupling two continuous linear elastic systems in parallel.

For the case of two homogeneous bars with distinct wave speeds, we derive the analytical expression of the dispersion relation and show that even though each individual bar has a linear dispersion relation and no band gaps, the coupled bars exhibit multiple band gaps with clear resonant frequencies within these gaps. The resonant frequencies may be periodic or quasi-periodic depending on whether of the ratio between the wave speeds is rational or irrational, respectively. This provides a significant improvement over prior designs that were limited to a single resonant frequency.

Our elastodynamic simulations confirm the analytical dispersion relation predictions and show an enhanced attenuation effect for time domain disturbances when the frequency of excitation is close to one of the local resonant frequencies. Since the resonant frequency yields infinite imaginary part of the wave number, it produces the fastest attenuation effect of frequencies within the band gap and thus is an ideal way of suppressing wave propagation in a medium without viscous dissipation. Compared to tuned mass dampers^31^, our design provides effective vibration mitigation at multiple frequencies and larger flexibility in controlling the width of the band gaps due to the possibility of adjusting a larger number of parameters.

Our results suggest that more efficient wave control scenarios may emerge if heterogeneous bars are coupled instead of homogenous bars. A heterogeneous bar may possess its own band gap and thus after coupling with another heterogeneous bar, we have found that multiple resonant frequencies appear along with more band gaps. We further give an example in the Supplementary Information that coupling multiple bars in parallel preserve the resonant response and may even lead to lower frequency band gaps. This opens new pathways for designing more complicated structures, assembled from simpler components, with numerous resonant frequencies and adjustable band gap response.

We also show the dependence of attenuation strength on the compliance of the coupling constraint. Theoretically, the divergence of the imaginary part of wave number at specific frequencies is only achieved in the limit when the stiffness of the coupling constraint is infinite. However, for practical purposes, this limit is achieved if the stiffness of the coupling constraint is high enough. In particular, we have shown that if the two bars are weakly coupled, the dispersion relation of the composite system approaches the linear dispersion relation of each bar individually. As the coupling strength increases, band gaps develop, and the imaginary part of the wavenumber at the characteristic resonant frequencies increases as well. Thus, the response of the system is tunable by the strength of coupling which may be controlled during the manufacturing process.

There are different ways that may be envisioned to practically realize the coupling between parallel bars as examined in this paper. This may include, for example, coupling using magnetic attachments^32^, ultrasonic welding^33^, or mechanical couplers^34^. The choice of the specific coupling technique may depend on the nature of materials to be joined (e.g. polymeric vs metallic) as well as the required strength of coupling. As shown here, the attenuation strength within the band gaps may be tunable by changing the stiffness of the coupling constraint providing a direct tool for evaluating implications of different manufacturing techniques on the system response. This topic is of special interest to the authors, and is currently being explored in collaboration with experts in manufacturing and experimental testing.

A simple device for modulating the elastic wave is shown in this paper. We demonstrated that by periodically coupling just a part of a homogeneous bar with another homogeneous/composite bar, we may significantly modulate wave amplitude in the through-going bar. The frequencies within the band gap are largely attenuated after passing the coupled region. This provides a shielding effect for the continuing bar beyond the region of coupling and suggests an effective way for vibration mitigation and control in existing structures using elastic attachments.

Furthermore, inspired by the structure of the dispersion relation of the coupled elastic bars, we present for the first time, to the best of our knowledge, a realization of a simple mechanical chopper. In the electronics literature, a chopper is an electronic switch that is used to interrupt a signal under the influence of another one. Our simple device in Fig. 8 may be designed to achieve a similar effect for mechanical waves. By selecting the length and the material properties of the attachment bar, we can generate a specific phase shift between the signal passing within the through-going bar and the attached bar, such that when the two signals superpose as they emerge out of the end of the coupled region, most of the outgoing signal is annihilated. We show that for a given material choice, the length of the attachment may be chosen to chop a target frequency. By making the attachment bar telescopic and the connection between the two bars mobile or detachable through magnetic forces or dynamic reversible bonding, we may be able to design a simple but tunable 1D wave modulation device that may target multiple frequencies. This finding is particularly relevant for designing against fatigue. For a structure under fatigue loading, as the number of cycles increases, the bar may fail eventually by fatigue according to Paris’ law^35^. However, when we introduce the computed phase shift and the chopper effect, we may allow only the first half sine wave to pass even if its amplitude is comparable to the incident one, but we eliminate all the other cycles and thus may significantly increase the operating lifetime.

Future extension of this work will explore both the applications of these design ideas in controlling wave propagation in 2D and 3D systems as well as hybrid bar-plate systems for manipulating, attenuating, and directing wave motions. The models described here also open new opportunities for vibration control in existing structures and material systems through simple modifications of the system architecture (i.e. adding short elastic attachments). This may be particularly relevant for lattice materials and bracing systems in structural engineering applications. This is a topic of an ongoing investigation for the authors.

## Methods

The elastodynamic response of finite coupled bars under harmonic loading is simulated by the Finite Element Method (FEM)^36^. The bar is discretized using two-node linear elements. Newmark Beta method with $\beta =1/4$ is used as the time marching algorithm with constant average acceleration.

### Modal representation of a homogeneous bar

The governing equation of a single infinite bar with longitudinal motion is

7 $$\frac{{\partial }^{2}u}{\partial {t}^{2}}={c}^{2}\frac{{\partial }^{2}u}{\partial {x}^{2}}$$

in absence of body force, where *u* is the longitudinal displacement, *x* is the axis coordinates, $c=\sqrt{EA/\rho }$ is the longitudinal wave speed, *E* is the Young’s modulus, *A* is the area of section, and $\rho$ is mass density per unit length.

Consider a steady state solution with the form

8 $$u({\rm{x}},{\rm{t}})=U({\rm{x}}){{\rm{e}}}^{-i\omega t}$$

where $\omega$ is the oscillation frequency. Substituting (8) in (7), we obtain

9 $$\frac{{d}^{2}U}{d{x}^{2}}+{(\frac{\omega }{c})}^{2}U=0$$

where *U* is the mode shape corresponding to the given frequency ω. The general solution of equation (9) is given by

10 $$U={A}_{1}\,\cos (\frac{\omega }{c}{\rm{x}})+{A}_{2}\,\sin (\frac{\omega }{c}{\rm{x}})$$

where *A*_1_ and *A*_2_ are constant coefficients satisfying the boundary conditions.

### Dispersion relation of coupled parallel bars

We consider two homogeneous bars connected as shown in Fig. 1. It follows from equation (10) that the displacements in each bar are given by:

11 $$\begin{array}{c}{U}_{1}({\rm{x}})={A}_{1}\,\cos (\frac{\omega }{{c}_{1}}{\rm{x}})+{A}_{2}\,\sin (\frac{\omega }{{c}_{1}}{\rm{x}})\\ {U}_{2}({\rm{x}})={A}_{3}\,\cos (\frac{\omega }{{c}_{2}}{\rm{x}})+{A}_{4}\,\sin (\frac{\omega }{{c}_{2}}{\rm{x}})\end{array}$$

where $x\in [0,{\rm{a}}]$, ${u}_{i}({\rm{x}},{\rm{t}})={U}_{i}({\rm{x}}){{\rm{e}}}^{-i\omega t}$, and ${c}_{i}$ is the wave speed for bar $i=1,2$. Since the two bars are constrained at $x=0$ and $x=a$, we have

12 $$\begin{array}{c}{U}_{1}(0)={U}_{2}(0)\\ {U}_{1}(a)={U}_{2}(a)\end{array}$$

By applying the Bloch wave condition^37,38^, we have

13 $$U(0)=U(a){{\rm{e}}}^{-iqa}$$

14 $${\rm{\Sigma }}(0)={\rm{\Sigma }}(a){{\rm{e}}}^{-iqa}$$

where $U(0)={U}_{1}(0)={U}_{2}(0)$, $U(a)={U}_{1}(a)={U}_{2}(a)$, ${\rm{\Sigma }}(0)={{\rm{\Sigma }}}_{1}(0){+{\rm{\Sigma }}}_{2}(0)$, ${\rm{\Sigma }}(a)={{\rm{\Sigma }}}_{1}(a){+{\rm{\Sigma }}}_{2}(a)$, ${\rm{\Sigma }}(x)$ follows from the decomposition of stress field $\sigma ({\rm{x}},{\rm{t}})={\rm{\Sigma }}({\rm{x}}){{\rm{e}}}^{-i\omega t}$, and $q$ is the wave number. For the case of a homogeneous bar $\sigma =EA\frac{\partial u}{\partial x}$. This leads to

15 $${\rm{\Sigma }}(x)=EA\frac{dU(x)}{dx}$$

Substituting equations (11) and (15) into equations (12)–(14) and simplifying the four equations, we can write the four equations in a matrix form as

16 $${\bf{Dv}}=0$$

where $${\bf{v}}={({A}_{1},{A}_{2},{A}_{3},{A}_{4})}^{{\rm{T}}}$$ and

$${\bf{D}}=(\begin{array}{cccc}1 & 0 & -1 & 0\\ \cos (\omega a/{{\rm{c}}}_{1}) & \sin (\omega a/{{\rm{c}}}_{1}) & -\cos (\omega a/{{\rm{c}}}_{2}) & -\sin (\omega a/{{\rm{c}}}_{2})\\ 1-{e}^{-iqa}\,\cos (\omega a/{{\rm{c}}}_{1}) & -{e}^{-iqa}\,\sin (\omega a/{{\rm{c}}}_{1}) & 0 & 0\\ {\rho }_{1}{c}_{1}\omega {e}^{-iqa}\,\sin (\omega a/{{\rm{c}}}_{1}) & {\rho }_{1}{c}_{1}\omega (1-{e}^{-iqa}\,\cos (\omega a/{{\rm{c}}}_{1})) & {\rho }_{2}{c}_{2}\omega {e}^{-iqa}\,\sin (\omega a/{{\rm{c}}}_{2}) & {\rho }_{2}{c}_{2}\omega (1-{e}^{-iqa}\cos (\omega a/{{\rm{c}}}_{2}))\end{array})$$

In order for **v** to have a non-trivial solution, we must have:

17 $${\rm{\det }}({\bf{D}})=0$$

Similar procedure may be applied to compute dispersion relation of other cases mentioned in this paper.

### Coupling of two bars each has more than one material phase

For coupled two composite bars, assume the two bars are composites containing *n*_1_ and *n*_2_ kinds of material. There are $2({n}_{1}+{n}_{2})$ unknowns in the expression of $U({\rm{x}})$ in total so that ${\bf{v}}={({A}_{1}\cdots {A}_{2({n}_{1}+{n}_{2})})}^{{\rm{T}}}$. In addition to four equations from equations (12)–(14), we have $2({n}_{1}+{n}_{2}-2)$ equations from continuity at the ${n}_{1}+{n}_{2}-2$ interfaces and $2({n}_{1}+{n}_{2})$ equations in total. The dispersion relation is then computed by solving equation (10) with the $2({n}_{1}+{n}_{2})\times 2({n}_{1}+{n}_{2})$ matrix **D**.

### Coupling of two bars with finite stiffness constraint

In this case, we may choose the unit cell as shown in Fig. 6 with the coupling point at its center. Assume *U*_1_(x), *U*_2_(x), *U*_3_(x), *U*_4_(x) representing the mode shape of left half of bar1, right half of bar1, left half of bar 2, and right part of bar 2 respectively. There are 8 unknowns associated with those four mode shapes. Bloch wave condition gives 4 equations for displacement and stress of 2 bars as in equations (13),(14). Continuity of displacement within each bar at the connection location gives 2 additional equations as

18 $$\begin{array}{c}{U}_{1}(a/2)={U}_{2}(a/2)\\ {U}_{3}(a/2)={U}_{4}(a/2).\end{array}$$

Equilibrium of stress at the constraint location gives 2 more equations:

19 $$\begin{array}{c}{{\rm{\Sigma }}}_{1}(a/2)={{\rm{\Sigma }}}_{1}(a/2)+k[{U}_{3}(a/2)-{U}_{1}(a/2)]\\ {{\rm{\Sigma }}}_{3}(a/2)={{\rm{\Sigma }}}_{4}(a/2)+k[{U}_{1}(a/2)-{U}_{3}(a/2)].\end{array}$$

Thus, matrix **D** may be formed as before and the dispersion relation may be computed by applying equation (17).

## Electronic supplementary material

Coupling of three bars
