# Supplementary material for: Emergent wave phenomena in coupled elastic bars: from extreme attenuation to realization of elastodynamic switches
Source: Sci Rep. 2017 Nov 24;7:16204. doi: 10.1038/s41598-017-16364-8 (PMC5701181; doi:10.1038/s41598-017-16364-8)
Supplement: Supplementary file 1 — Coupling of three bars [file 41598_2017_16364_MOESM1_ESM.doc]

**Emergent wave phenomena in coupled elastic bars: from extreme attenuation to realization of elastodynamic switches**

**Qianli Chen^1^,*, and Ahmed Elbanna^1^**

**^1^Department of Civil and Environmental Engineering, University of Illinois at Urbana Champaign**

***Correspondence to qchen35@illinois.edu**

**Supplementary Information**

**Coupling of three bars.** For the coupled three bars, we have 2 more unknowns than the coupled two homogeneous bars case. Assuming the mode shape of the third bar is $U_{3}(\mathrm{x})$, we have 2 more equations as

| $U_{n}(0)=U_{n-1}(0)$ $U_{n}(a)=U_{n-1}(a)$ | (S1) |
| --- | --- |

where n = 3 here. Following the same procedure outlined in Methods section, the dispersion relation of three periodically coupled homogeneous is given by

| $\cos\left(qa\right)=$ $\frac{z_{1}\text{cos}\left(\theta_{1}\right)\text{sin}\left(\theta_{2}\right)\text{sin}\left(\theta_{3}\right)+z_{2}\text{cos}\left(\theta_{2}\right)\text{sin}\left(\theta_{1}\right)\text{sin}\left(\theta_{3}\right)+z_{3}\text{cos}\left(\theta_{3}\right)\text{sin}\left(\theta_{1}\right)\text{sin}\left(\theta_{2}\right)}{z_{1}\text{sin}\left(\theta_{2}\right)\text{sin}\left(\theta_{3}\right)+z_{2}\text{sin}\left(\theta_{1}\right)\text{sin}\left(\theta_{3}\right)+z_{3}\text{sin}\left(\theta_{1}\right)\text{sin}\left(\theta_{2}\right)}$ | (S2) |
| --- | --- |

where $\theta_{i} =\omega a/c_{i},i=1,2,3$.

The imaginary part of wave number solutions are given in Fig. S1 where $a=1$, $\rho_{1}=\rho_{2}=\rho_{3}=1$, $(EA)_{1}=(EA)_{2}/4=(EA)_{3}/9=1$. For comparison, the cases of two bar coupling for bars 1&2, 1&3, 2&3 are also shown here. Within the frequency domain from 0 to 12, the resonant frequencies for case 1&2, 1&3, and 2&3 are $\omega=3.64,8.93$, $\omega=3.46,5.97$, and $\omega=7.21$respectively while the coupled three bars case has resonant frequencies $\omega=3.33,7.00$. We note that the coupled three bars case has a lower resonant frequency than its coupling components while the other resonant frequency is close to its combination of stiffer sub-systems. Extreme attenuation occurs at frequencies corresponding to the zeros of the denominator of the dispersion relation.


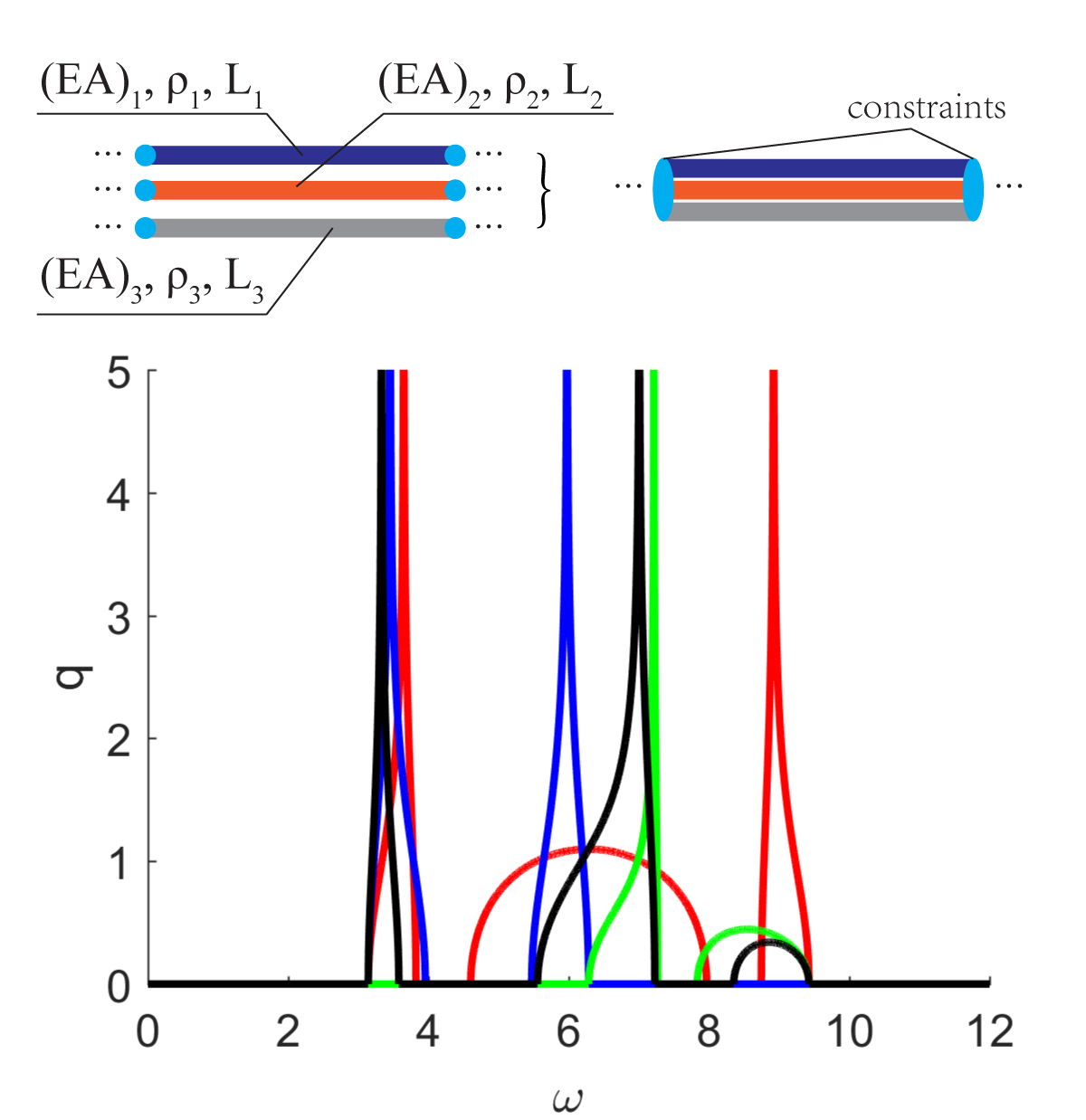


Fig. S1 Imaginary part of the solution of dispersion relation (bottom) and unit cells (top) of coupled three bars [$a=1$, $\rho_{1}=\rho_{2}=\rho_{3}=1$, $(EA)_{1}=(EA)_{2}/4=(EA)_{3}/9=1$, red: coupled 1&2; blue: coupled 1&3; green: coupled 2&3; black: coupled three bars;]
